# The Russian-Ukraine conflict, mental health, and Covid-19: A triad of concerns for children residing within the conflict zone

**DOI:** 10.1016/j.amsu.2022.104815

**Published:** 2022-11-05

**Authors:** Ashraf Mahmoud, Agathon Kimario, Jonaviva Anthony, Erick Magese, Emmanuel Ezaka, Anastasiia Volkova, Goodluck Nchasi

**Affiliations:** Faculty of Medicine, Kilimanjaro Christian Medical University College, Kilimanjaro, Tanzania; Department of Medicine, Catholic University of Health and Allied Science, Mwanza, Tanzania; Department of Psychology, University of Nigeria, Nsukka, Nigeria; Faculty of Medicine, Lviv National Medical University, Ukraine; Department of Medicine, Catholic University of Health and Allied Science, Mwanza, Tanzania

**Keywords:** Conflict, Mental health, COVID-19, Children

## 1 Introduction

Conflicts may be extremely traumatizing since they disrupt the existing mental tranquility. The most recent conflict, which is being debated globally, is between Russia and Ukraine. Conflicts have an impact on people's mental health because people react differently to conflicts depending on their intensity [1]. Even though mental health concerns are prevalent in the contemporary generation, individuals rarely contemplate their significance [2]. Mental disorders can be found in any population, but they are more prevalent in conflict-affected communities due to conflict-related stress [3]. This commentary aims to demonstrate how the conflict between Russia and Ukraine has hampered mental health amidst the COVID-19 pandemic, particularly among children, and provides essential recommendations for supporting children and victims of similar conflicts.

## 2 The Russian-Ukraine conflict

**Fig. 1:**
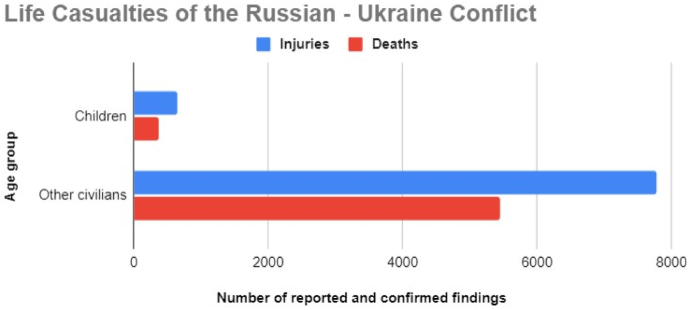
Shows the estimates of the life casualties of the Russian- Ukraine conflict as per the report from the Office of the United Nations High Commissioner for Human Rights (OHCHR) as of September 11, 2022 [4].

Russia and Ukraine have been in conflict for more than 5 years over Ukraine's numerous jurisdictions. Russia attacked Ukraine on February 24th, intensifying an already-existing pandemic. As a result, there have been casualties and significant property damage; on August 22, 2022, the Office of the United Nations High Commissioner for Human Rights (OHCHR) confirmed 13,477 civilian casualties, 41% of which were reported as civilian deaths, though the actual number of casualties may be higher [4]. Furthermore, as of September 11th, 375 children had been killed and another 647 had been injured, as shown in Fig. 1, with more expected to be killed either deliberately or not [4]. The conflict has also affected immunization programs for infectious diseases in children, as seen by 44% of polio vaccination coverage in Ukraine [5].

## 3 Mental health concerns

Emotional, psychological, and social well-being are the fundamental components of mental health. Exposure to conflict increases the likelihood of children developing mental health disorders such as anxiety and depression [4]. Given the rise in depression and anxiety, not all children will be traumatized, but they may react differently to stressful situations or events [6]. In terms of mental health, someone may simply look at a child and assume that he or she is doing well [6]. Furthermore, as the conflict progresses, children experience severe consequences that damage both their health and well-being; millions of them are subjected to the horrors of war, including missiles, home destruction, bloodied individuals, unsuccessful evacuation, family separation, and having to flee death [4].

## 4 COVID-19

Ukraine, like other countries, is still dealing with the Coronavirus disease (COVID-19), with 4,949,283 confirmed cases and 107,779 fatalities. As of February 27, 2022, however, 31,668,577 vaccination doses have been delivered [7]. At a greater expense, children are at a much-increased risk of mental health damage. This is because the tension, fear, and despair that existed during the COVID-19 era have become even more pronounced in the present environment [7]. Children's stress and anxiety have increased massively throughout the conflict compared to the COVID-19 era.

## 5 Concerns, efforts, and recommendations

Primary care, screening, and immunization programs for several illnesses are being jeopardized. This demonstrates that individuals, particularly children, suffering from chronic conditions such as diabetes and heart failure would be unable to access adequate care or health services. Because there are currently no screening programs, certain pediatric cancers may be overlooked or detected too late. Infectious diseases are also more likely to spread when clean water is scarce, and the sanitation system is weakened. Inadequate immunization programs, which result in poor vaccination coverage, raise the probability of outbreaks of vaccine-preventable illnesses such as measles and polio, both of which have a negative impact on children's mental health [8].

Several attempts have been made to aid children who are suffering because of the ongoing Russian-Ukraine war, particularly during this difficult period of the COVID-19 pandemic.

On the one hand, the United Nations International Children's Emergency Fund (UNICEF) has adopted several programs to assist victims and children in Ukraine. It has supplied basic critical necessities to victims, such as personal protective equipment for health personnel to shield them from COVID-19 while responding to victims' needs, as well as medicine and first aid kits [9]. Furthermore, UNICEF has been collaborating with administrators to ensure that children receive prompt assistance^4^. The purpose of the supporting mobile teams is to guarantee that children, particularly those who feel persistently insecure, get protection services and psychological care [9].

On the other hand, children's psychologists have advised caregivers to use different methods and tactics which might be useful when having conversations with children during this challenging time.

1. **Need to make time and allow the child to speak as long as the child wants to:** Give children enough room to speak what they perceive about conflict, so take time to listen to what they think, saw and heard [10].
2. **Narrate the conversation to the child:** A child needs to hear from you about your perception of the conflict but be careful not to over-explain the scenario as it may bring unnecessary anxiety [10].
3. **Substantiate their feelings**: It is equally important to support the children in conversation and avoid the feeling that will make them feel ignored [10].
4. **Guarantee them that the world is working hard to settle the conflict**: Children need to be told that the current situation is not their fault so they should not stop doing things that make them happy like playing and visiting friends [10].
5. **Allow them to make a practical contribution when wanting to help**: Many children would wish to help those affected by the war, by creating a fundraising initiative or producing art influencing peace. So, these children need to be supported [10].

Further, there is a need to share education with the general population on how to deal with difficult and stressful mental problems [11]. Also, the mass media can be of immense importance to spreading mental health promotion messages to the community [11].

## Ethical approval

Ethical approval is not applicable for commentary submission.

## Sources of funding

We did not receive any funding pertaining to the completion of this work.

## Author contribution

Conceptualization: **Ashraf Mahmoud, Goodluck Nchasi**.

Writing – review and editing: **All authors**.

Final approval of manuscript: **All authors have read and approved the final version of the manuscript**.

## Registration of research studies

1. Name of the registry: Not Applicable.

2. Unique Identifying number or registration ID: Not Applicable.

3. Hyperlink to your specific registration (must be publicly accessible and will be checked): Not Applicable.

## Consent

Consent is not applicable for commentary submission as there was no data collection.

## Guarantor

Ashraf Mahmoud.

Kilimanjaro Christian Medical University College.

Kilimanjaro Tanzania.

Email: ashyhms@gmail.com.

## Declaration of competing interest

No conflict of interest is to be declared within this submission.

## References

[1] Virga D., CurSeu P.L., Maricutoiu L., Sava F.A., Macsinga I., Magurean S. (2014). Personality, relationshipconflict, and teamwork-related mental models. PLoS ONE9.

[2] Ezaka E., Asiegbu F., Chibuike O. (2022). Impact of social support on mental wellbeing among internet users in Nigeria during COVID-19 pandemic. Neurol Neurosc.

[3] Baingana F. (2003). https://www.researchgate.net/publication/277785598_Mental_Health_and_Conflict.

[4] (2022). Ukraine Civilian War Casualties.

[5] Schlein L. (2022). https://www.voanews.com/a/who-war-interrupts-routine-lifesaving-immunizations-in-ukraine/6547218.html.

[6] Kekatos M. Mental health effects of Ukraine war zone on children. https://abcnews.go.com/International/mental-health-effects-ukraine-war-zone-children/story?id=83203801&msclkid=e9af42a5acfe11ec973dc0d1bcb8b598.

[7] WHO Regional Office for Europe (2022). https://worldhealthorg.shinyapps.io/EURO_COVID-19_vaccine_monitor/.

[8] Ukraine: war has an impact on people's health beyond bullets and. https://theconversation.com/ukraine-war-has-an-impact-on-peoples-health-beyond-bullets-and-bombs-178062.

[9] UNICEF (2022). https://www.unicef.org/emergencies/war-ukraine-pose-immediate-threat-children.

[10] (2022, February 25). Save the children. Ukraine: 5 ways to talk to children about conflict. https://www.savethechildren.net/news/ukraine-5-ways-talk-children-about-conflict-0.

[11] Mollica R.F. (2004).

